# NEAR: NEW OPPORTUNITIES FOR AGING RESEARCH

**DOI:** 10.1093/geroni/igac059.964

**Published:** 2022-12-20

**Authors:** Debora Rizzuto, Scott Hofer

## Abstract

The global increase in life expectancy is one of the greatest achievements of the last half century. However, the demographic developments towards an older population also challenge many parts of the society, especially the health care. Promoting healthy ageing is therefore one of the most important commitments of the 21st century and to succeed, scientifically based knowledge of older individuals’ health- and care requirements are needed. To better understand the individual and population aging process, the National E-infrastructure for Aging Research (NEAR) was founded in 2018 to build and run a national infrastructure by integrating existing databases from the 15 major longitudinal studies on aging and health in Sweden. To show the added value of NEAR, this symposium will present results from four ongoing NEAR projects: 1) Developing a metric of global brain integrity in multiple Swedish studies with different scanners; 2) Functional aging trajectories and drug interactions; 3) Long-term prediction of dementia using machine learning algorithms; 4) The new aging – how different aspect of ageing has changed over half a century. The creation of national infrastructures is needed to achieve broad, multidisciplinary research perspectives that cannot be achieved by individual databases. Moreover, to address the increased health demands of an older population and enhance new opportunities for aging research, a critical mass of data is needed to increase sample sizes, variations, representativeness, and generalizability. Ultimately, this can lead to the identification of sustainable intervention strategies for better health and care for older persons during the coming decades.

